# An Unusual Presentation of Eisenmenger Syndrome in a Middle-Aged Woman Without Known Cardiac History

**DOI:** 10.7759/cureus.34668

**Published:** 2023-02-06

**Authors:** Howard N Rainey, Alison W LePera

**Affiliations:** 1 Internal Medicine, Edward Via College of Osteopathic Medicine, Blacksburg, USA; 2 Emergency Medicine, Edward Via College of Osteopathic Medicine, Blacksburg, USA

**Keywords:** atrial septal defects, right-sided heart failure, severe pulmonary arterial hypertension, right to left shunting, eisenmenger syndrome

## Abstract

Eisenmenger syndrome (ES) is a severe cardiac complication that arises from an untreated congenital cardiac defect, leading to the reversal of shunt flow, pulmonary hypertension, and cyanosis. This uncommon complication most frequently arises from small ventricular septal defects that are undiagnosed due to a lack of symptoms. However, it may arise from the reversal of any left-to-right cardiac shunt. In the following report, we present a case of acute-onset ES in a 52-year-old woman with no past cardiac history. The patient presented to the emergency department with a clinical presentation consistent with likely pulmonary embolism; however, after extensive work-up, this etiology of respiratory failure was deemed incorrect. After rapid respiratory decline requiring mechanical ventilation, the medical team performed two transthoracic echocardiograms (one with agitated saline study), one transesophageal echocardiogram, and a right cardiac catheterization on the patient. This work-up revealed pulmonary hypertension, right heart failure, and an atrial septal defect. Given these findings, the work-up was suggestive of ES secondary to an atrial septal defect shunt reversal. Because of the complexity of treatment, the patient was transferred via air to a university tertiary medical institution for extracorporeal membrane oxygenation along with other advanced treatments.

This case provides a framework for the clinical presentation and treatment of this life-threatening disease. We hope that this information will help providers understand the clinical presentation, work-up, treatment, and prognosis of patients with Eisenmenger syndrome.

## Introduction

Eisenmenger syndrome (ES) is a medical phenomenon first described in 1897 in which an untreated congenital left-to-right cardiac shunt reverses into a right-to-left shunt [1]. The shunt reversal is caused by increased pulmonary and right heart pressure from long-term shunting from the high-pressured left heart [2]. The resulting effects of this reversal are generally recognized as a triad, including elevated pulmonary vascular resistance, systemic-to-pulmonary congenital cardiovascular communications, and cyanosis [3]. Though any left-to-right shunt can lead to ES, the most common occurrence is seen in patients with small ventricular septal defects (VSD); this is most often the defect leading to ES because the diagnosis of a small VSD can be missed due to lack of symptoms [4,5]. Fortunately, given the astronomical advances in modern medicine, particularly in surgically managing neonatal cardiac shunts, ES has become quite rare with a decreasing incidence from 2.5 per million individuals per year in 1977 to 0.2 per million individuals per year in 2012 [6]. The importance of early detection and treatment in ES cannot be overlooked, particularly to combat an adjusted 10-year mortality rate that has ranged between 30% to 40% for the last 70 years [7]. Below, we report a case of a 52-year-old female with no cardiac history who developed rapid-onset atrial septal shunt reversal, resulting in right heart failure and pulmonary hypertension. Because of the rarity of this disease in modern medicine, this report can be a valuable source of information for other medical professionals that must treat this condition.

## Case presentation

**Figure 1:**
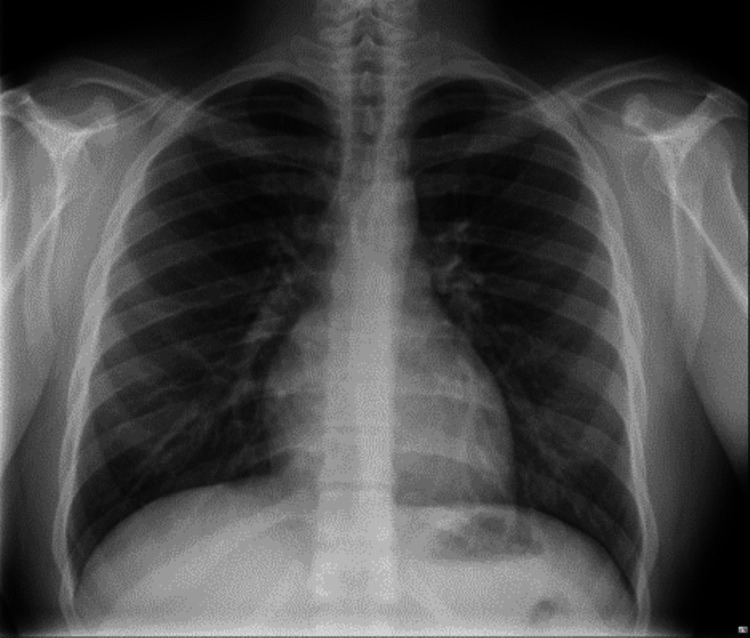
Initial chest X-ray from the emergency department. No acute findings were reported.

**Figure 2:**
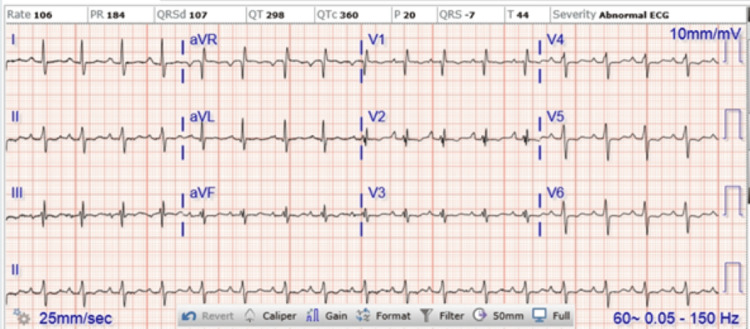
Patient's electrocardiogram while in the emergency room. This reveals sinus tachycardia and a left axis deviation. No sign of acute ischemia.

The patient of interest is a 52-year-old, Caucasian female non-smoker with a medical history of hypertension and obesity who presented to the hospital with complaints of lower back pain, left leg pain, and shortness of breath that had been progressively worsening since being involved in a motorcycle accident approximately one month prior to arrival. On initial arrival at the emergency department, the patient was tachycardic and tachypneic with an oxygen saturation of 89% on three liters of oxygen via nasal cannula. In addition, she rated her left leg pain as 10 out of 10. On auscultation, the patient’s heart sounded normal, and her lungs were clear to auscultation bilaterally. To rule out pulmonary embolism (PE), a computed tomography angiogram (CTA) with a contrast of the chest was ordered that was non-revealing. A left lower extremity ultrasound was also ordered to assess for deep vein thrombosis (DVT) which, too, was non-revealing. The X-Rays of the left foot, left tibia and fibula, lumbar spine, and chest (Figure 1) were also employed with no signs of injuries. On lab work, the patient had an initial troponin of 0.04 ng/mL and brain natriuretic peptide (BNP) of 804 ng/mL; however, the remainder of the labs were within normal limits at this time. Initial electrocardiogram (ECG) showed sinus tachycardia with left axis deviation; no sign of myocardial infarction (Figure 2).

The patient was admitted to the medical/surgical unit with telemetry under the hospitalist’s service. The initial diagnoses for this patient were an acute hypoxic respiratory failure, elevated troponin level, and left lower extremity cellulitis. On admission to the hospitalist’s service, the patient was now on six liters of oxygen with an oxygen saturation of 87%. The lungs remained clear to auscultation bilaterally with no auscultatory signs of pathology. The initial plan reported by the admission team was to order a transthoracic echocardiogram (TTE), continue to trend the patient’s troponin, treat left lower extremity cellulitis with Rocephin, treat possible DVT and/or PE with Lovenox, manage pain, and provide supplemental oxygen.

**Figure 3:**
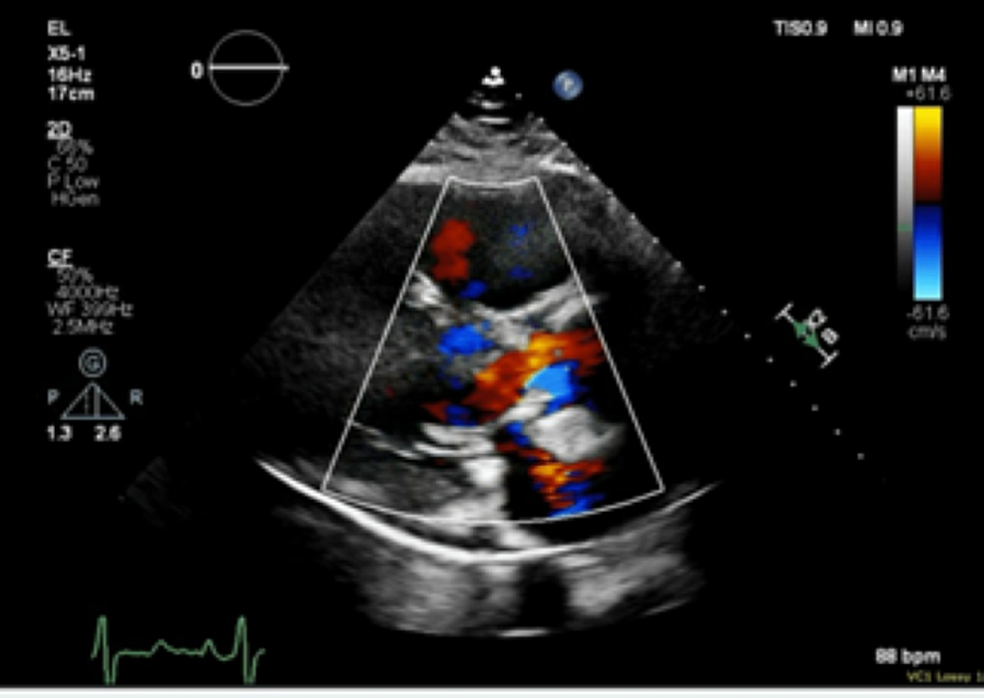
Snapshot image from the immediate TTE TTE: Transthoracic echocardiogram

During the first night of admission, the patient’s tachypnea significantly progressed, requiring 15 liters of oxygen via high-flow nasal cannula. The patient received an immediate TTE to identify the potential etiology of respiratory failure. The TTE revealed the following findings: severe concentric left ventricular hypertrophy with an ejection fraction estimated at 55% to 60%, severely enlarged right ventricle with reduced systolic function, severely dilated right atrium, mild aortic regurgitation, moderate mitral annular calcification, moderate tricuspid regurgitation, pulmonary regurgitation, and severe pulmonary hypertension (Figure 3). At this time, all suspected that the patient’s findings were consistent with a thromboembolic event; therefore, full anticoagulation was initiated. The patient’s troponin peaked at 0.05 ng/mL and trended downwards (the elevation was likely due to demand ischemia), and her BNP fell to 100 ng/mL.

**Figure 4:**
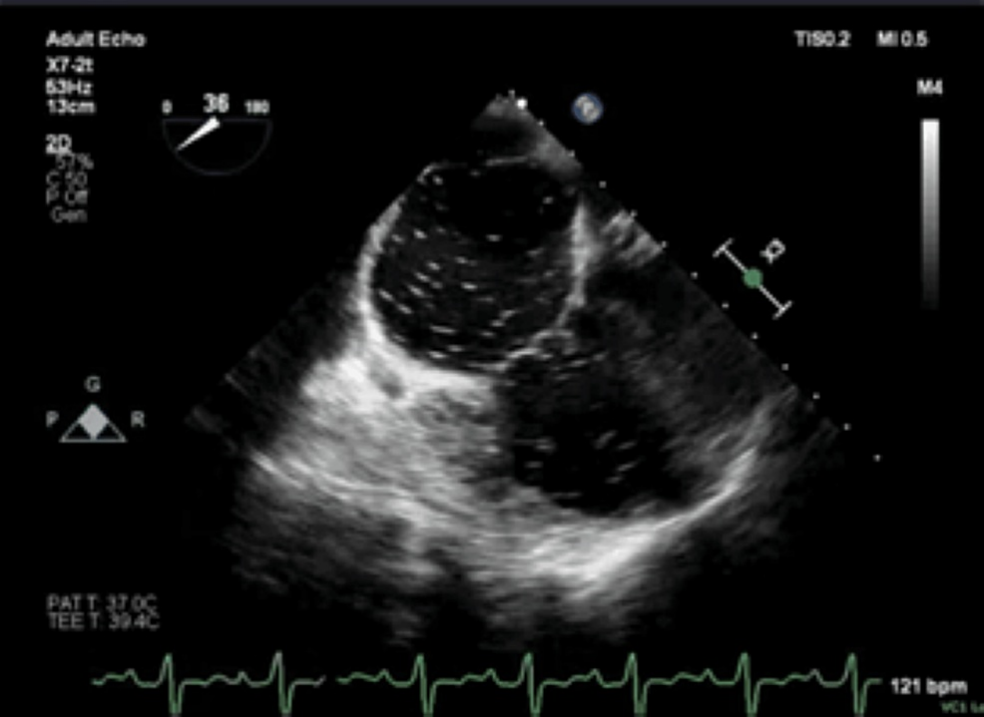
Second TTE with a successful agitated saline study revealing an interatrial septal defect with biatrial shunting TTE: Transthoracic echocardiogram

The following day, the patient had a repeat TTE given the lack of improvement. However, this time an agitated saline study (“bubble study”) was also performed (Figure 4). Agitated saline studies are often added to TTEs to assess for intracardiac versus intrapulmonary shunting [8]. On repeat, the results showed an interatrial septal defect with biatrial shunting of the agitated saline. Given these findings, a transesophageal echocardiogram (TEE) was ordered to further evaluate this suspicious finding. In addition, pulmonology recommended performing a ventilation/perfusion (V/Q) scan to assess for microthrombi, which was unrevealing. The patient’s respiratory situation continued to decline; she was now on 50 liters of oxygen with a 100% fraction of inspired oxygen (FiO2). Despite this, her oxygen saturation remained low at 87%. Epoprostenol was given to combat the patient’s severe pulmonary hypertension, which was not successful.

**Figure 5:**
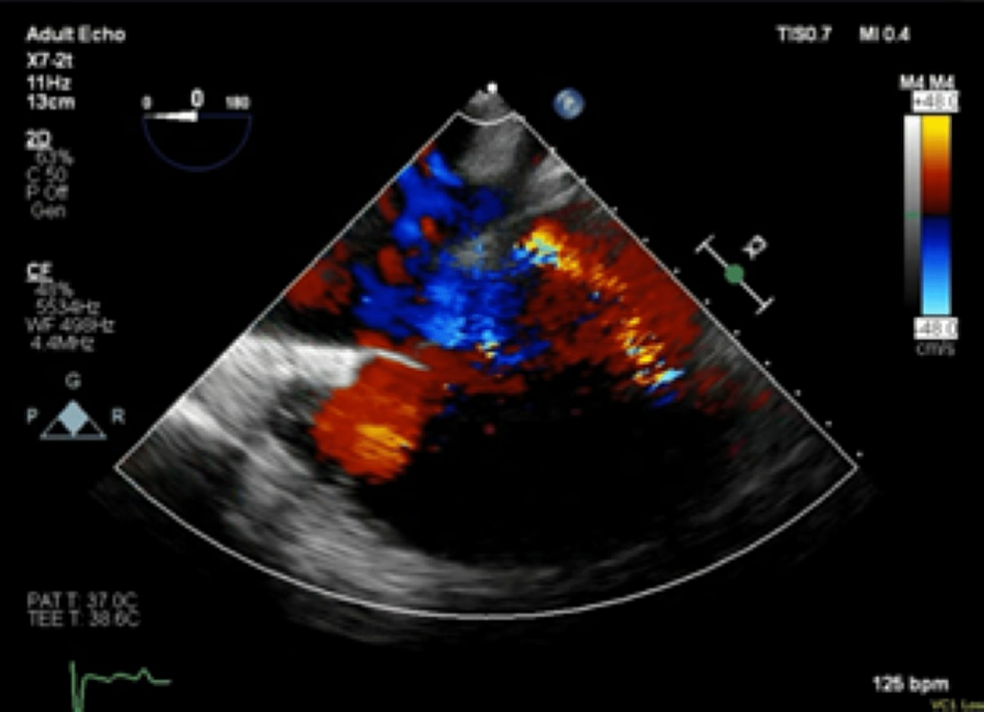
Transesophageal echocardiogram confirming the findings of biatrial cardiac shunting by the previous two TTE studies TTE: Transthoracic echocardiogram

Given the significant worsening of her respiratory capabilities, the critical care team intubated the patient prior to cardiology performing TEE. Once the patient was stable, TEE was performed. The TEE showed evidence of intracardiac right-to-left shunt by contrast injection, though the location of the shunt could not be completely elucidated (Figure 5). Given the worsening of the patient’s condition and the identification of a shunt with severe pulmonary hypertension, cardiology recommended that the patient be transferred to a tertiary care center for a more thorough evaluation and access to a facility with the means to treat this patient’s shunt reversal. As only 25.4% of shunts can be successfully diagnosed with echocardiogram alone [5], right heart catheterization (RHC) was performed which revealed a pulmonary artery pressure of 78 mmHg and pulmonary wedge pressure of 15 mmHg. This, coupled with the echocardiogram findings, was consistent with right heart failure secondary to reversed intra-cardiac shunt. The patient was diagnosed with Eisenmenger syndrome and transferred to a tertiary medical facility via air for extracorporeal membrane oxygenation (ECMO) and further advanced treatment.

## Discussion

The patient of interest with a past medical history of hypertension and obesity presented with right heart failure and pulmonary hypertension followed by the identification of a right to left cardiac shunt consistent with ES. Though there were certainly many characteristics of this case that were consistent with the classic manifestations of ES, there were several features of this specific case that made correctly diagnosing this patient quite challenging. To begin, the location of the cardiac defect was in the atrial septum. Based on previous studies, this is quite rare as most ES cases arise from an untreated VSD or patent ductus arteriosus (PDA) [9-11]. Per a study performed by Frank et al., 53% of ES cases arose from a preexisting VSD; only 13% of cases were associated with an atrial septal defect (ASD) [6]. Though an uncorrected ASD is more commonly diagnosed in adult-onset ES compared to pediatric cases [12], this was still highly unanticipated. However, the agitated saline test performed with the second TTE identified an interatrial septal defect with biatrial shunting, confirming this unexpected finding. This key finding reflects the importance of performing an agitated saline test while implementing echocardiography, particularly when suspicions of pulmonary hypertension arise.

Another intriguing aspect of this case is the age of the patient of interest. A study performed in 2013 by Hjortshoj et al. revealed that the average age of diagnosis for adult-onset ES is during the third decade of life [13]. In the case outlined above, the patient was 52 years old with no prior cardiac history. The patient likely had lived with an asymptomatic cardiac defect, which did not cause any symptoms or complaints while physiologically functioning as a left-to-right shunt. However, when the right heart pressure surpassed that of the left heart pressure from the shunt, the patient developed symptoms of right heart failure and pulmonary hypertension.

Though the patient’s treatment at the accepting medical center cannot be provided in this report, this information can be speculated based on first-line therapies and other studies with similar patient populations. Because of the patient’s severe respiratory and cardiovascular decline, the plan was to place the patient on ECMO while further treatments could be investigated. Treatment for patients with severe symptoms includes any of the following: endothelin receptor antagonist alone, endothelin receptor antagonist plus phosphodiesterase-5 (PDE-5) inhibitor, endothelin receptor antagonist plus guanylate cycle stimulator, or heart and lung transplant if the patient meets candidacy [14]. The treatment regimen likely included one of the former three treatments while awaiting candidacy for a heart and lung transplant. Despite the employment of any of the treatment regimens, the patient likely maintained a poor prognosis given the irreversible shunt that formed over several decades.

## Conclusions

Although ES is a disease that continues to become more uncommon with early detection of congenital heart diseases in neonates, this cardiac complication continues to provide a poor prognosis and high short-term mortality rate in affected individuals. In order to combat this unfortunate complication, early detection, and adequate treatment are paramount for all providers that encounter patients with pulmonary hypertension of cardiac etiology. As the medical community continues to understand pulmonary hypertension and ES, more life-sustaining treatments will hopefully become the first line for these conditions. With this report, the authors hope to provide other medical practitioners with an understanding of this complicated pathologic presentation so that in other cases it may be considered earlier in the disease course, yielding more thorough work-up and treatments. This is particularly important in this pathology as swift identification and treatment can improve prognosis, depending largely on cardiac pressure gradients that worsen over time. 
